# Modified Suction-Assisted Cartilage Shaver for Axillary Osmidrosis

**DOI:** 10.1155/2019/7314753

**Published:** 2019-05-08

**Authors:** Yu-Ju Tseng, Chih-Hung Lee, Shang-Hung Lin

**Affiliations:** ^1^Department of Dermatology, Kaohsiung Chang Gung Memorial Hospital and Chang Gung University College of Medicine, Kaohsiung 833, Taiwan; ^2^Chang Gung University of Science and Technology, Chiayi Campus, Chiayi City 613, Taiwan

## Abstract

Apocrine osmidrosis (AO) is a chronic, recurrent, and disturbing disease characterized by malodorous secretion from apocrine glands. Despite various conservative and nonsurgical treatments, surgical removal of apocrine glands remains the cornerstone for AO treatment. Conventional suction-assisted cartilage shaver is effective; however, there are several risks and complications. Hence, we modified the conventional method to achieve better effectiveness and reduce complications. This paper aims to evaluate the clinical effectiveness and the complications arising from the modified suction-assisted cartilage shaver for AO. Thirty-nine patients (M/F=11/28, average age 26.3 years) received this surgical treatment for AO from 2013 to 2017 in the Department of Dermatology at Kaohsiung Chang Gung Memorial Hospital, Taiwan. A suction-assisted cartilage shaver was introduced for the ultimate removal of the subcutaneous tissue containing the apocrine glands. A 0.5 cm incision was made in the center of the identified elliptical surgical area at each axilla. After defatting, the incision was closed primarily. The defatting skin was anchored to the axillary fascia by using 4-0 sutures without drains. We then evaluated the clinical efficacy and complications. The mean duration of follow-up was 31.8 months (12–68 months). Among patients receiving the modified cartilage shaving for AO, 92.3% achieved excellent-to-good results, 5.1% had acceptable results, and 2.6% had fair results. None of them experienced poor clinical efficacy. There was no skin necrosis, hematoma, nor wound infection after the surgery. There were no recurrences in all these patients 2 years after the surgery. This modified suction-assisted cartilage shaver for AO results in good efficacy, a low complication rate, and a low recurrence rate. The method is superior to the conventional one due to tissue glue-free procedure, greater comfort in postoperative care, minimal wounds, less hematoma, and less skin necrosis. The clinical study registration number of this study is NCT03793374.

## 1. Introduction

Apocrine osmidrosis (AO) is a chronic and disturbing apocrine disease characterized by malodorous secretion in the axillary area, which often bothers patients of both genders in different aspects, including their relationships, social interactions, and their careers. The pathogenesis of AO may involve the interaction between the bacteria, the pheromones, the 5*α*-reductase, and the abnormal* ABCC11* gene [1].

Several conservative managements have been introduced to patients with AO, such as topical antiperspirants and botulinum toxin injection, both of which only provided the temporary symptom relief, but recurrence is very common [2]. Microwave-based treatment is the latest noninvasive method that provides satisfactory outcomes; however, it takes several serial treatments to achieve optimal results. The efficacy of the microwave-based treatment is 72.5–90% after 1-year follow-up, which is inferior to surgical methods of over 90% efficacy [3–5]. Moreover, several complications of brachial plexus injury, leading to mild-to-severe numbness and muscle weakness, were reported [6–8].

Surgical removal of the apocrine glands is a conventional procedure, which provides a sustained effect on AO management. Various techniques, from the excision of subcutaneous tissues [14, 15], curettage, and ultrasonic liposuction [16, 17] to laser-assisted methods [18, 19], have been introduced to the surgical management of AO. The subepidermal excision by scissors provides a high rate of efficacy, up to 96.8%, but it also causes large surgical wounds and leads to up to 33.9% epidermal necrosis [20]. The more aggressive removal of apocrine glands leads to better outcomes, but it is also associated with a higher risk of developing complications, such as hematomas, seromas, infections, poor wound healing, and scar formation. With the progress of minimal invasive intervention and combination therapy in recent years, wound size could be smaller and the rates of common complications after AO surgery have been tremendously decreased to less than 4.5% (1.5%-4.5%) [21]. Therefore, in terms of efficacy and acceptable risks, surgical intervention remains the leading choice for managing AO.

To remove apocrine glands of axillae, the cartilage shaver provides a delicate method with less wounding in contrast to the conventional surgery [10, 12, 13]. However, the results of the minimal invasive method regarding the risk of hematoma formation, infection, and wound necrosis remain elusive. In our study, we introduced a modified suction-assisted cartilage shaver for AO patients and evaluated the efficacy and safety. We also compared our modified method to other cartilage shaver methods and microwave-based methods for more comprehensive information.

## 2. Materials and Methods

### 2.1. Patients

After obtaining the agreement of the Chang Gung Medical Foundation Institutional Review Board (ID: 201801926B0), we retrospectively included patients from July 2013 to September 2017. There were 39 patients, who were treated for AO by the same dermatologist with suction-assisted cartilage shaver, at the Department of Dermatology in Kaohsiung Chang Gung Memorial Hospital, Taiwan. There were 28 females and 11 males, ranging from 14 to 54 years of age. They were all affected by axillary osmidrosis, which had a detrimental impact on their daily life due to the embarrassing odor. Operations were performed under local anaesthesia and on an outpatient basis.

### 2.2. Operation

During the procedure, the patient's axillae were exposed with the patient lying supine and the arms abducted to avoid injury to the brachial plexus. We cut the axillary hair short for better visualization of the region of the apocrine glands, which are located near the hair follicles. The hair-bearing elliptical region of axillae was marked before cutting the hair. Tumescent solution was prepared with 0.1% lidocaine, 1:500000 epinephrine and 10 mEq/L sodium bicarbonate. We injected the tumescent solution into the subcutaneous level of each axilla in view of the hydrodissection ability of tumescent solution and for minimizing the bleeding.

**Figure 1:**
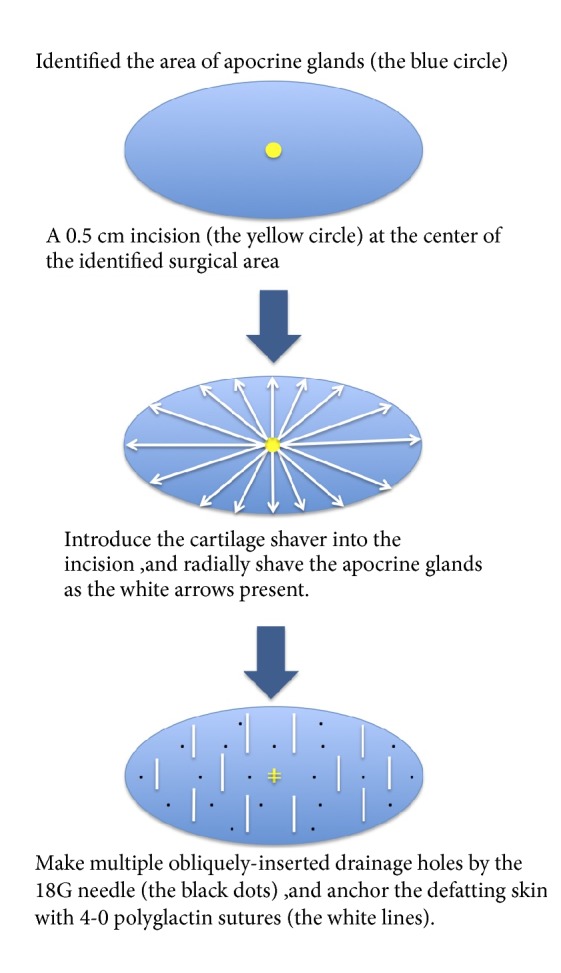
Blue ellipses represent the identified surgical area of the apocrine glands. We firstly make a 0.5 cm incision in the center of the area (the yellow circle), dissect the subcutaneous tissue, and then introduce the cartilage shaver from the incision. Apocrine glands at dermal-subcutaneous junction are removed radially as the white arrows indicate. The incision wound is closed primarily with 2 stitches of 4-0 polyglactin (yellow lines in the center), and there are multiple obliquely inserted drainage holes made by the 18G needle, on the defatting flap (the black dots). Finally, we anchor the defatting skin with 4-0 polyglactin sutures, which are parallel to the axillary skin crease (the white lines).

**Figure 2:**
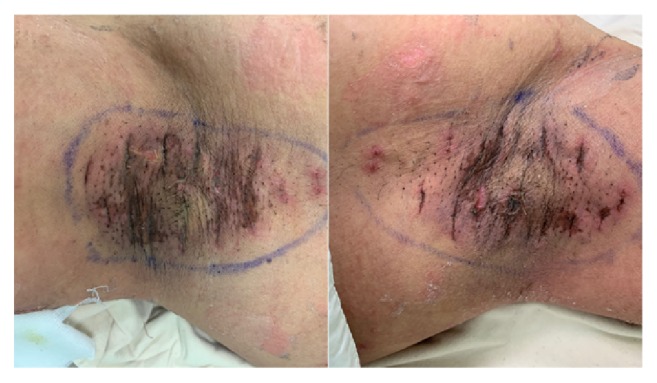
Clinical pictures in a 26-year-old male patient before removing the stitches 7 days after the operation. The left picture is his left axilla while the right one is his right axilla.

A 0.5 cm long incision was made in the center of the identified elliptical surgical area at each axilla for the easier arthroscopic access to remove the apocrine glands at the dermo-subcutaneous junction and to hide the scar in the skin crease. A suction-assisted cartilage shaver (E9005 System, Linvatec Corporation, Largo, Florida, USA) was introduced through the incisions to remove the subcutaneous tissue containing the apocrine glands radially (Figure 1). We set the system to keep the inner cannula at 1500 rotations per minute in oscillation mode. After defatting, the incisions were closed primarily with 4-0 polyglactin. We anchored the defatting skin to the axillary fascia by using 4-0 polyglactin sutures instead of the tie-over dressing used in the conventional shaver procedure, and we also made several drainage holes by inserting an 18G needle obliquely into the defatting skin rather than placing the drainage tubes used in the conventional shaver procedure. Therefore, drainage tubes were no longer needed. We removed the stitches 7 days after the operation (Figure 2).

### 2.3. Efficacy Assessments

The patients' medical history, physical examinations, and vital signs were carefully collected before the operation. We retrospectively evaluated pre- and postoperative clinical efficacy with a patient-centered scoring method. The severity of AO before the operation was classified from 1 to 5 to indicate the least severe to the most severe condition, from undetectable, mild, moderate, and severe to unbearable malodor. The clinical efficacy was classified using 5 grades: poor (0–20%), fair (21–39%), acceptable (40– 59%), good (60–79%), and excellent (80–100%), which was evaluated based on the elimination of malodor and postoperative satisfaction. To evaluate the safety, adverse complications, such as hematoma, seroma, infection, wound necrosis, skin necrosis or perforation, and scar formation, were recorded.

## 3. Results

We included thirty-nine patients, with a mean age of 26.3 years old (from 14 to 54 years old). There were 11 male patients with an average age of 23 years and 28 female patients with an average age of 27.6 years. The mean duration of follow-up after the operation was 31.8 months (from 12 to 68 months) (Table 1). Before the operation, 64.1% of our patients ranked between 4 and 5 for the severity of AO, and the percentage was similar in both gender groups (63.6% in the male group and 64.2% in the female group). In the elimination of malodor, out of the total of 39 patients, 36 (92.3%) expressed positive satisfaction of the procedure, 2 (5.1%) had acceptable results, and 1 (2.6%) had fair results (Table 1). None of them had poor clinical efficacy. There was no skin necrosis, hematomas, nor wound dehiscence reported 1 month after surgery. One patient complained about obvious scar formation at unilateral axilla. None of our patients had recurrence 2 years after the surgery. Besides common complications after AO surgery, there were 7 out of 39 patients having mild compensatory hyperhidrosis in the upper trunk, which did not disturb patients' daily life (Table 1).

There were 10 adolescent patients, including 9 females and 1 male, with an average age of 17 years, and the mean duration of follow-up was 34.6 months. In this group, 90% (9/10) of them reported good-to-excellent clinical efficacy. Further, except for one patient who had unilateral keloid formation after the operation, no patients reported compensatory hyperhidrosis.

## 4. Discussion

The apocrine glands at the junction of dermis and subcutaneous tissues were very close to the hair follicles. Contrary to eccrine glands, which cover almost all keratinized skin surfaces of human body, the apocrine glands exist in specific areas, such as the axillae, areolae and nipples, ear canal, eyelids, nostril wings, and the external genitalia. The apocrine glands produce odoriferous sticky secretions to serve as the pheromones, which function as sexual attractants, territorial markers, and warning signals in mammals. Physiologically the apocrine glands reach maturity after puberty. However, some people experience axillary osmidrosis (AO), which takes form of excessive apocrine secretions and intolerable malodor.

The history of surgical treatment for AO can be dated back to 1962 [22]. There is no gold standard method for AO treatment. The management of AO depends on the severity of AO and the evaluation of the pros and cons of different methods. We summarize the comparison of AO management in Table 2. Despite various methods having been introduced to the management of AO [2, 6, 7, 14–18], surgical intervention remains the most effective way of completely eliminating the apocrine glands for the patients with moderate-to-severe AO [21]. A systemic review and meta-analysis in 2017 compared the efficacy and the safety among different treatment modalities associated with AO [21]. In terms of efficacy, surgery has the lowest recurrence rate, compared with liposuction and laser treatment [21]. Regarding safety, evaluated by incidence of hematoma formation or skin necrosis, surgery was second to liposuction [21]. During the process of extensively removing the apocrine glands in order to achieve better clinical outcomes, various complications could occur, such as hematoma, seroma, skin infection, wound necrosis, skin necrosis or perforation, and scar formation. Therefore, with advances in surgical techniques, safety is the major concern in the management of AO. Decades of effort have been given to the field of surgical treatment for AO, to improve both clinical efficacy and the patients' safety. In 1977, Inaba et al. first introduced the ‘tissue shaver' as a curette to manage AO, which provided a timesaving operation with effective results [23, 24]. Lee et al. also introduced suction-assisted cartilage shaver to 82 patients in 2004, and they had 92% of satisfaction results from the patients and no major complications within 3 months after operation [10]. We modified the procedure by using a single incision in the center of the hairy part of the axillae, placing the shaver into the incision, and shaving the subcutaneous fat tissues radially. The defatting flaps were anchored by sutures to enhance flap stabilization and were penetrated by several needle holes to facilitate drainage (Figure 1). Major complications of hematoma and skin necrosis resulted from poor attachment of the defatting flap to the tissues underneath and the accumulation of discharge between the defatted flap and the fat underneath. Traditionally the problem is managed by using tie-over dressing and placing the drainage tubes in such a way as to promote better circulation of the defatting flap, which is effective, although it leads to much discomfort during the postoperative period due to simultaneous immobilization at the same time. By using our method, tie-over dressing and drainage tubes are no longer required, leading to an improvement of comfort during the recovering period. Our patients also benefit from the free of use of the tissue glues, which reduces the financial burden to patients. Also, they experience greater comfort during the postoperative period without sacrificing safety. In fact, none of our patients experienced hematoma, skin necrosis or wound infection during the six-year retrospective study.

Compared with other studies using cartilage shaver for AO (Table 3), our modified method provides a noninferior clinical efficacy and relatively low complication rates. The microwave-based method for AO or axillary hyperhidrosis has been introduced in recent years. There is currently no systemic meta-analysis data to compare the efficacy of AO treatment between surgery and the microwave-based method. Therefore, we also searched the literature for microwave-based treatments for hyperhidrosis (Table 3). Although the microwave-based treatment is a noninvasive procedure, it also bears the risk of local anaesthesia before the procedure, as the surgical procedures do. The clinical efficacy of the microwave method is no better than the cartilage shaver group, and there are much higher variable degrees of short-term and long-term complications, including pain, soreness, swelling, and burn injury, after the microwave-based procedure. These complications alter the sensation in the treatment limb and result in skin nodulation in the long-term follow-up [5–7]. Most importantly, we have the longest duration of follow-up compared with other cartilage shaver studies and microwave studies, which arguably supports the argument that our modified method provides good sustainability for the treatment of AO.

The apocrine glands do not function physiologically until puberty. The affecting patients of AO are mostly young adults. However, 25.6% (10/39) of our patients were adolescents at the time of receiving the surgery. All of these young patients had a good response, and the efficacy persisted for at least 1 year. The speed of maturation of apocrine glands varied between individuals and some may have had AO early in their adolescent years. Therefore, age should not become a barrier to have surgery for young patients suffering AO.

In our study, we also found an interesting phenomenon—that 17.9% of patients report compensatory hyperhidrosis mainly in the upper trunk and scalp after surgery—which has never been mentioned in previous literature about surgical removal of axillary apocrine glands. To our knowledge, compensatory hyperhidrosis was frequently observed in patients receiving sympathectomy for axillary osmidrosis or hyperhidrosis [25]. The range of compensatory hyperhidrosis in sympathectomy-treated patients varied, and there were up to 50% of patients, who received the sympathectomy, having compensatory hyperhidrosis postoperatively in Lee et al.'s study [25]. The exact mechanisms of compensatory hyperhidrosis in patients who received surgical removal of axillary apocrine glands are not clear. We speculate that some of the patients might pay more attention to their sweating condition after the AO surgery, so they might have a misconception about the sweating. However, further investigations are required.

There are several advantages of our modified surgical method. The subcutaneous tissue excision for AO sometimes leads to poor wound healing, scar formation, or peripheral hematoma accumulation. The single incision with multiple small stabbing wounds achieves a good drainage function to avoid hematoma formation. The site of the central incision wound is placed at the skin crease of the armpit, so the operative scar is usually not evident. Another problem that might be encountered when performing the subcutaneous shaving for AO is the difficulty in eliminating the apocrine glands around the incision wound completely. This is also the reason that most surgeons suggest using two incisions to facilitate the elimination of apocrine glands. However, if the wound is placed at the center of the armpit, we can still easily eliminate the apocrine glands around the incision by pinching and everting the skin to visualize the glands.

There are also a few disadvantages. First, it has longer learning curve for the physicians to achieve optimal results. Besides, well-distributed stabbing wounds should be made meticulously otherwise the drainage function might be impaired.

The main limitation of our study is that there is still no objective evaluation for quantifying the severity of AO, which is currently the universal limitation in all AO literature. The patient-centered numerical method is a quick and easy way to approach AO patients. Psychological factors contributed most to a patient's decision to undergo the surgery. The number of female patients is more than twice that of the male patients in our study, which may explain why AO has a greater psychosocial impact on female patients.

In our study, 92.3% of patients had a satisfactory response, 5.6% of patients had acceptable feedback, and only 1 patient felt that there were no differences after surgery (Table 1). No additional tissue glues were needed in our study. There was no hematoma or seroma formation, nor wound infection, keloid formation, or other severe complications noted 1 month after the surgery. The benefits of the AO surgery could last for at least 2 years, and the longest disease-free period is 5 years.

## 5. Conclusions

Surgical intervention for AO has been associated with variable complications. This modified suction-assisted cartilage shaver for AO results in good efficacy, low complication rates, and low recurrence rates in both adolescent and adult patients.

## Figures and Tables

**Table 1:** Patient demographics and clinical results (n=39).

Variables	Results (%)
*Age (years)*	14 – 54 (Average 26.3)
Male	23
Female	27.6
*Number of patients (mean age)*	
Male	11/39 (28.2)
Female	28/39 (71.8)
*Duration of follow-up (months)*	12 – 68 (Average 31.1)
*Clinical efficacy*	
Excellent	17/39 (43.6)
Good	19/39 (48.7)
Acceptable	2/39 (5.1)
Fair	1/39 (2.6)
Poor	0 (0)
*Postoperative complication (axillae)*	
Hematoma / seroma	0/78 (0)
Defatted skin necrosis or perforation	0/78 (0)
Wound edge necrosis	0/78 (0)
Wound infection	0/78 (0)
Scar formation	1/78 (1.3)
*Compensatory hyperhidrosis*	7/39 (17.9)
*Adolescent patients (n=10)*	
* Number of male/female (mean age)*	1/9 (17)
* Duration of follow-up (months)*	12 – 68 (Average 34.6)
* Clinical efficacy*	
Excellent	3/10 (30)
Good	6/10 (60)
Acceptable	1/10 (10)
Fair	0 (0)
Poor	0 (0)
*Postoperative complication (axillae)*	1 keloid formation at unilateral axilla
*Compensatory hyperhidrosis*	1/10 (10)

**Table 2:** Comparison of different methods in axillary osmidrosis (AO) management.

	Methods	Advantages	Disadvantages	Indicated patients
Conservative management	Topical antiperspirants	(i) Noninvasive (ii) Easy to use	(i) Temporary effects (ii) Poor efficacy	Mild to moderate AO patients
	Botulinum toxin injection	(i) Good efficacy (ii) Noninvasive	(i) Need periodic use to maintain the efficacy (ii) Pain during the injection (iii) High cost	

Invasive management (to remove the apocrine glands)	Direct excision of dermis and subcutaneous tissues ± skin excision	(i) Good efficacy (ii) Under local anaesthesia	(i) Large wound (ii) Discomforts during postoperative periods (iii) Higher rates of hematoma formation, skin necrosis, keloid formation, and wound infection	Moderate to severe AO patients
	Curettage	(i) Moderate to good efficacy (ii) Smaller wound and less painful than direct excision (iii) Under local anaesthesia	(i) Complications depend on the physician experience	
	Ultrasonic liposuction	(i) Moderate to good efficacy (ii) Small incision wound (iii) Short recovery time (iv) Under local anaesthesia	(i) Complications depend on the physician experience	
	Laser-assisted	(i) Moderate to good efficacy (ii) Small incision wound (iii) Short recovery time (iv) Under local anaesthesia	(i) Higher recurrence rate than liposuction and surgery (ii) Higher complication incidence than the liposuction and surgery	
	Microwave-based	(i) Good efficacy (ii) No surgical wound (iii) Short learning curve (iv) Under local anaesthesia	(i) Require multiple sessions to achieve better results (ii) Postoperative pain, swelling, and numbness (could be temporary) (iii) Possibility to injure the brachial nerve (iv) High cost	
	Suction-assisted cartilage shaver	(i) Good efficacy (ii) Under local anaesthesia (iii) Better removal of the apocrine glands	(i) Long learning curve (ii) Complications depend on the physician experience	

Sympathectomy	Upper thoracic sympathectomy	(i) Moderate to good efficacy	(i) Need general anaesthesia (ii) Compensatory hyperhidrosis (iii) Possibility of nerve injury (iv) Risk of pneumohemothorax

**Table 3:** Comparison of cartilage shaver and microwave device for AO.

Author	Methods for AO	Patients numbers	Efficacy (%)^a^	Complications (%)	Wound healing	Follow-up duration
Tung [9], 2001	1 cm incision, cartilage shaver	64	91.4	3.9 (wound edge necrosis)	5 days	6-13 months, mean 9.3 months
Lee et al [10], 2005	Two 1 cm incisions, cartilage shaver	89	91.2	1.1 (hematoma and skin perforation) 0.6 (of wounds)	21 days	14-28 months, mean 20 months
Wu [11], 2007	0.5–1.0 cm incision, cartilage shaver	156	92.3	7.7 (wound edge necrosis)	21 days	6-59 months, mean 16 months
Chern et al [12], 2011	0.8 cm incision, cartilage shaver	30	94	1.7 (wound local infection)	7 days	3-13 months, mean 10 months
Hsu et al [13], 2018	1 cm incision, cartilage shaver	19	94	3.8% (ecchymosis)	Not mentioned	3 months
Current study, 2018	0.5 cm incision, cartilage shaver	39	92.3	1.3% (scar formation)	7 days	12-69 months, mean 31 months

Glaser et al [3], 2012	Microwave	120	89%	28%		12 months
Hong et al [4], 2012	Microwave	31	90.3%	26% - 71% temporary adverse effects		12 months
Scuderi et al [5], 2016	Microwave	20	72.5%	25% nodular formation		Mean 5 months
Chang et al [8], 2017	Microwave	1	-	Median nerve neuropathy affected both sensory and motor function		6 months (partially recovered sensory and motor function

^a^ Elimination of malodor, rated as excellent to good.

## Data Availability

The data used to support the findings of this study are available from the corresponding author upon request.

## References

[1] Nakano M., Miwa N., Hirano A., Yoshiura K.-I., Niikawa N. (2009). A strong association of axillary osmidrosis with the wet earwax type determined by genotyping of the ABCC11 gene. *BMC Genetics*.

[2] Heckmann M. (2003). Amelioration of body odor after intracutaneous axillary injection of botulinum toxin A. *JAMA Dermatology*.

[3] Anna Glaser D., Coleman W. P., Fan L. K. (2012). A randomized, blinded clinical evaluation of a novel microwave device for treating axillary hyperhidrosis: the dermatologic reduction in underarm perspiration study. *Dermatologic Surgery*.

[4] Hong C. H., Lupin M., O'Shaughnessy K. F. (2012). Clinical evaluation of a microwave device for treating axillary hyperhidrosis. *Dermatologic Surgery*.

[5] Scuderi S., Manoharan P., Lim D., Manoharan S. (2017). A survey of patient satisfaction with use of microwave device for axillary hyperhidrosis. *Australasian Journal of Dermatology*.

[6] Hsu T., Chen Y., Tu Y., Li C. (2017). A systematic review of microwave-based therapy for axillary hyperhidrosis. *Journal of Cosmetic and Laser Therapy*.

[7] Lee S., Chang K., Suh D., Song K., Ryu H. J. (2013). The efficacy of a microwave device for treating axillary hyperhidrosis and osmidrosis in Asians: a preliminary study. *Journal of Cosmetic and Laser Therapy*.

[8] Chang C., Chen C., Hsu K. (2017). Brachial plexus injury after microwave-based treatment for axillary hyperhidrosis and osmidrosis. *Journal of Cosmetic and Laser Therapy*.

[9] Tung T. (2001). Endoscopic Shaver With Liposuction for Treatment of Axillary Osmidrosis. *Annals of Plastic Surgery*.

[10] Lee J. C., Kuo H., Chen C., Juan W., Hong H., Yang C. (2005). Treatment for axillary osmidrosis with suction-assisted cartilage shaver. *British Journal of Plastic Surgery*.

[11] Wu W. (2009). Ablation of apocrine glands with the use of a suction-assisted cartilage shaver for treatment of axillary osmidrosis. *Annals of Plastic Surgery*.

[12] Chern E., Yau D., Chuang F., Wu W. (2010). Arthroscopic shaver with refinement for axillary osmidrosis. *International Journal of Dermatology*.

[13] Hsu K.-C., Wang K.-Y. (2018). Sparing subcutaneous septa avoids skin necrosis in the treatment of axillary bromhidrosis with suction-curettage shaving. *Journal of Cosmetic Dermatology*.

[14] Yoshikata R., Yanai A., Takei T., Shionome H. (1990). Surgical treatment of axillary osmidrosis. *British Journal of Plastic Surgery*.

[15] Tung T., Wei F. (1997). Excision of subcutaneous tissue for the treatment of axillary osmidrosis. *British Journal of Plastic Surgery*.

[16] Ou L.-F., Yan R.-S., Chen I.-C., Tang Y.-W. (1998). Treatment of axillary bromhidrosis with superficial liposuction. *Plastic and Reconstructive Surgery*.

[17] Chung S., Yoo W., Park Y., Shin K., Park B. (2000). Ultrasonic surgical aspiration with endoscopic confirmation for osmidrosis. *British Journal of Plastic Surgery*.

[18] Jeong J. H., Hong J. M., Pak C. S., Kim J. H., Heo C. Y. (2014). Treatment of axillary osmidrosis using a laser with a 1,444-nm wavelength. *Dermatologic Surgery*.

[19] Lee S. G., Ryu H. J., Kim I. (2014). Minimally invasive surgery for axillary osmidrosis using a combination of subcutaneous tissue removal and a 1,444-nm Nd:YAG laser. *Annals of Dermatology*.

[20] Qian J., Wang X. (2006). Radical treatment of axillary osmidrosis by subdermal excision of apocrine glands: a prospective study in 31 cases. *Journal of Plastic, Reconstructive & Aesthetic Surgery*.

[21] Shin J. Y., Roh S. G., Lee N. H., Yang K. M. (2017). Osmidrosis treatment approaches: a systematic review and meta-analysis. *Annals of Plastic Surgery*.

[22] Skoog T., Thyresson N. (1962). Hyperhidrosis of the axillae. A method of surgical treatment. *Acta chirurgica Scandinavica*.

[23] Inaba M., Ezaki T. (1977). New instrument for hircismus and hyperhidrosis operation: subcutaneous tissue shaver. *Plastic and Reconstructive Surgery*.

[24] Inaba M., Anthony J., Ezaki T. (1978). Radical operation to stop axillary odor and hyperhidrosis. *Plastic and Reconstructive Surgery*.

[25] Lee H., Chen C., Lee W., Chuang H., Kao M. (2008). Axillary hyperhidrosis and osmidrosis treated by ultrasonic surgical aspiration compared with transthoracic endoscopic sympathectomy. *World Neurosurgery*.

